# Thalamic Microstructural Alterations as Revealed by the T1/T2 Ratio in Chronic Pain Patients

**DOI:** 10.3390/jcm14092888

**Published:** 2025-04-22

**Authors:** Max van Grinsven, Richard Witkam, Erkan Kurt, Sezai Özkan, Anja van der Kolk, Kris Vissers, Dylan Henssen

**Affiliations:** 1 Department of Anaesthesiology, Pain and Palliative Medicine, Radboud University Medical Center, 6525 EZ Nijmegen, The Netherlands; max.vangrinsven@radboudumc.nl (M.v.G.); jesper.witkam@radboudumc.nl (R.W.); sezai.ozkan@radboudumc.nl (S.Ö.); kris.vissers@radboudumc.nl (K.V.); 2 Department of Medical Imaging, Radboud University Medical Center, 6525 EZ Nijmegen, The Netherlands; anja.vanderkolk@radboudumc.nl; 3 Department of Neurosurgery, Radboud University Medical Center, 6525 EZ Nijmegen, The Netherlands; erkan.kurt@radboudumc.nl; 4 Department of Nuclear Medicine, University Hospital Leipzig, 04103 Leipzig, Germany

**Keywords:** chronic pain, biomarkers, MRI, T1-weighted/T2-weighted ratio, PSPS-II, central post-stroke pain, microstructure

## Abstract

**Background/Objectives:** Neuroimaging biomarkers could offer more objective measures of the pain experience. This study investigated rT1/T2 maps of the brain as a novel biomarker for chronic pain in patients with central post-stroke pain (PSP) and persistent spinal pain syndrome type 2 (PSPS-II). **Methods:** Patients with PSP and PSPS-II were retrospectively included alongside healthy controls. Bias correction and intensity normalization were applied to the T1-weighted and T2-weighted images to generate the rT1/T2 maps of the brain. Subsequently, rT1/T2 maps were spatially correlated with neurotransmitter atlases derived from molecular imaging. **Results:** In total, 15 PSPS-II patients, 11 PSP patients, and 18 healthy controls were included. No significant differences between patient and control demographics were found. Significant decreases in rT1/T2 signal intensity (*p* < 0.001) were observed in the dorsal and medial part of the thalamus, left caudate nucleus, cuneus, superior frontal gyrus, and dorsal cervicomedullary junction in PSP patients. No significant changes were found in rT1/T2 signal intensity in PSPS-II patients. Significant correlations were found with CB1-, 5HT2a-, and mGluR5-receptor maps (pFDR = 0.003, 0.030, and 0.030, respectively) for the PSP patients and with CB1-, 5HT1a-, 5HT2a-, KappaOp-, and mGluR5-receptor maps (pFDR = 0.003, 0.002, 0.002, 0.003, and 0.002, respectively) in PSPS-II patients. **Conclusions:** These findings suggest that microstructural alterations occur in the thalamus, cuneus, and dorsal cervicomedullary junction in patients with PSP. The lack of significant findings in rT1/T2 in PSPS-II patients combined with the significant correlations with multiple neurotransmitter maps suggests varying degrees of microstructural deterioration in both chronic pain syndromes, although further research is warranted.

## 1. Introduction

Chronic pain remains a leading cause of disability and loss of quality of life, and hence imposes a significant burden on society considering its high global prevalence, ranging from 18 to 20.9 percent [1,2,3]. Given the elusive etiology and multifactorial and multidimensional nature of chronic pain, it remains notoriously difficult to find proper treatment strategies. Consequently, current treatment methods focus mostly on treating the symptoms of chronic pain rather than the underlying mechanism [4,5]. To address this gap, a recent call was made to define more objective biomarkers, as these could shed light on the etiology of chronic pain and provide more tangible measures of the pain experience [6]. Biomarkers could entail measurements like pulse and electromyography, but they can also be derived from neuroimaging techniques, such as magnetic resonance imaging (MRI) experiments [6].

Prior neuroimaging studies in chronic pain have identified various candidate biomarkers. Structural MRI studies have reported region-specific gray matter volume reductions, while diffusion tensor imaging has shown white matter tract changes associated with neuropathic pain [7,8]. Functional MRI findings of altered connectivity in pain-processing networks further underscore that chronic pain is accompanied by brain changes [7]. Despite these advances, no neuroimaging marker has yet been widely adopted in clinical practice. A promising new biomarker derived from MRI is the ratio of the T1-weighted (T1w) and T2-weighted (T2w) image intensity (rT1/T2), which has shown promising results in multiple sclerosis-related research and other diseases such as Alzheimer’s [9,10], schizophrenia [11], glioblastoma [12,13], and Huntington’s disease [14]. In 2011, Glasser et al. proposed this signal as a novel non-invasive biomarker for cortical myelin and tissue integrity [15]. Given that the T1w signal increases and the T2w signal decreases in myelin-rich regions, they hypothesized that dividing the T1w image by the T2w image results in a myelin-enhanced image [15]. Even though the rT1/T2 maps were initially conceived of as a measure of cortical myelin, histopathological validation studies failed to demonstrate a correlation with myelin density in postmortem specimens. Instead, these studies revealed neurite and dendrite density to be histopathological correlates of the rT1/T2 signal [16,17]. Additionally, other tissue compounds, including iron deposits, which can be a sign of active neuroinflammation, impact the rT1/T2 signal [18]. Consequently, the rT1/T2 maps can be perceived more broadly as a measure of microstructural tissue integrity and neuroinflammation. Another particular advantage of this method is its ability to mitigate scanner-related biases, as these biases consistently appear in both T1w and T2w images [15]. Moreover, because rT1/T2 maps can be generated retrospectively from standard clinical MRI scans without requiring additional sequences or specialized protocols, this technique represents a clinically feasible and cost-effective candidate biomarker. Finally, the development of available open-source research software allows the normalization of rT1/T2 maps, enabling groupwise comparisons [19].

rT1/T2 signal maps have not been studied in the context of chronic pain syndromes. In this study, we aimed to address the need for innovative biomarkers in patients with chronic pain by investigating rT1/T2 maps in two pain syndromes. The first group concerned central post-stroke pain (PSP) patients suffering from intractable central neuropathic pain. The second group consisted of patients suffering from persistent spinal pain syndrome type 2 (PSPS-II). Additionally, a control cohort of healthy subjects without pain was included. Additionally, to further explore the microstructural correlates of the rT1/T2 maps, they were spatially correlated with neurotransmitter maps derived from molecular imaging. By investigating two groups with distinct central origins, we aimed to assess whether patterns of microstructural alterations are shared or unique to a specific syndrome.

## 2. Results

### 2.1. Baseline Characteristics

In total, 15 PSPS-II patients and 11 PSP patients, both suffering from central neuropathic pain (CNP), were included. The control group consisted of 18 healthy subjects. All demographic data were normally distributed. The mean age at onset of the CNP in the PSPS-II and PSP groups was 57.9 years (±9.7 years) and 64.3 years (±12.5 years), respectively. Within the control group, the mean age was 60.5 years (±13.0 years). The PSPS-II group consisted of eight males, the PSP group of four, and the control group of nine. No statistically significant difference in age at onset of the CNP (*p* = 0.14), age compared to controls (PSP: *p* = 0.45; PSPS-II: *p* = 0.51), or sex distribution (*p* = 0.74) were found. Demographic characteristics are summarized in Table 1.

### 2.2. Whole-Brain Analysis Results

**Figure 1:**
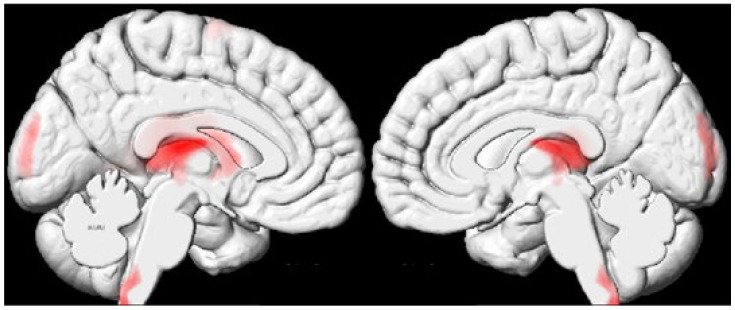
Significant clusters of rT1/T2 intensity differences between PSP patients and healthy controls, mapped on the ICBM152 template.

Whole-brain analysis revealed four clusters of significantly decreased rT1/T2 signal in the PSP group compared to healthy controls in the dorsal and medial part of the thalamus. Additionally, within the PSP group, the cuneus, superior frontal gyrus, dorsal cervicomedullary junction, and left caudate nucleus showed significant reductions in rT1/T2 signal (Figure 1). Peak and gravity MNI coordinates can be found in Table 2.

Four regions with significant rT1/T2 signal (*p* < 0.001) could be appreciated after cluster thresholding, including the dorsal and medial part of the thalamus (bilaterally), which are confluents of the left caudate nucleus, the cuneus (bilaterally), the left superior frontal gyrus, and the dorsal cervicomedullary junction/caudal medulla (bilaterally). Visualization was achieved using xjView (version 10.0).

No significant changes in rT1/T2 signal were found when comparing the PSPS-II group with controls. When comparing the PSPS-II patients with the PSP group, no clusters of significant alteration in rT1/T2 signal were observed.

### 2.3. Spatial Correlations with Neurotransmitter Maps

Post hoc spatial correlation analysis of the rT1/T2 maps of the PSP patients with multiple neurotransmitter maps revealed significant spatial correlations with the CB1 (ρ = 0.22; p_FDR_ = 0.003)-, 5HT2a (ρ = 0.19; p_FDR_ = 0.03)-, and mGluR5 (ρ = 0.27; p_FDR_ = 0.030)-receptor maps.

**Figure 2:**
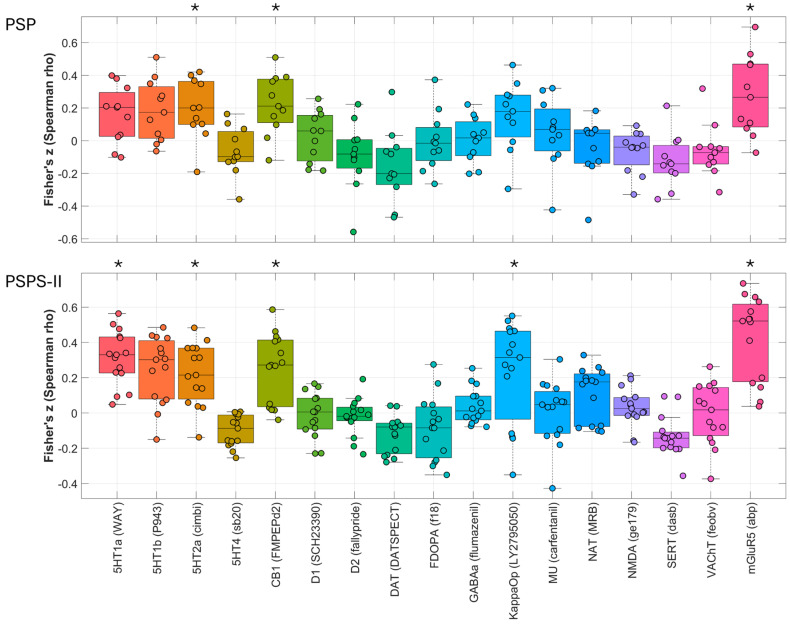
The spatial correlations between the PSP and PSPS-II rT1/T2 maps z-scored to the controls and the PET atlases measured in Spearman’s rho. * Significant correlation after FDR correction. Spatial correlation analysis of the rT1/T2 maps of the PSP patients with multiple neurotransmitter maps revealed significant spatial correlations with the CB1 (ρ = 0.22; p_FDR_ = 0.003), 5HT2a (ρ = 0.19; p_FDR_ = 0.03)- and mGluR5 (ρ = 0.27; p_FDR_ = 0.030)-receptor maps. Additionally, the rT1/T2 maps of the PSPS-II patients correlated with CB1 (ρ = 0.25; p_FDR_= 0.003)-, 5HT1a (ρ = 0.31; p_FDR_ = 0.002)-, 5HT2a (ρ = 0.22; p_FDR_ = 0.002)-, KappaOp (ρ = 0.23; p_FDR_ = 0.003)-, and mGluR5 (ρ = 0.43; p_FDR_ = 0.002)-receptor atlases.

Additionally, the rT1/T2 maps of the PSPS-II correlated with CB1 (ρ = 0.25; p_FDR_ = 0.003)-, 5HT1a (ρ = 0.31; p_FDR_ = 0.002)-, 5HT2a (ρ = 0.22; p_FDR_ = 0.002)-, KappaOp (ρ = 0.23; p_FDR_ = 0.003)-, and mGluR5 (ρ = 0.43; p_FDR_ = 0.002)-receptor atlases (Figure 2 and Table 3).

## 3. Discussion

In this study, clusters overlapping with the dorsal and medial thalamus, the left caudate nucleus, cuneus, the superior frontal gyrus, and the dorsal cervicomedullary junction showed a significantly decreased T1w/T2w-ratio in patients suffering from PSP when compared to controls without chronic pain. These clusters disappeared when the PSP group was compared with the PSPS-II group. Furthermore, no significant differences in the T1w/T2w ratio were found when comparing the PSPS-II group with controls. Despite the initial perception that rT1/T2 maps measure myelin density, histopathological studies have instead shown a correlation with dendrite density (estimated effect = [0.21, 0.84]) and neurite density (β = 3.464 × 10^−2^ per 10^2^ neurites/mm^2^) [16,17]. Additionally, iron increases the rT1/T2 signal by reducing T2w signal intensity [18,20]. The discussion will be guided by these biological correlates.

### 3.1. Decreased Microstructural Integrity of the Thalamus

The decreased signal of part of the thalamus on rT1/T2 maps can be explained by decreased thalamic microstructure in PSP patients. This is the first study investigating cerebral microstructural changes using rT1/T2 maps in chronic pain syndromes. However, one previous study examined microstructural changes in the brain using diffusion-weighted imaging. In this study, decreased fractional anisotropy was found in the spinothalamic tract and superior thalamic radiation in central post-stroke pain patients [8]. This decrease in white matter fractional anisotropy is believed to reflect decreased white matter integrity [21]. This report of decreased microstructural integrity of the superior thalamic radiation partially corroborates the results of the present study of a decrease in thalamic microstructure. However, the superior thalamic radiation sprouts from the ventral thalamic nuclear group [22], whereas our study found alterations in the dorsal and medial thalamic nuclear groups. Furthermore, a previous study found significantly lower ADC values in the spinothalamic tract of patients suffering from PSPS-II [23], implying neuroplastic changes in this large afferent thalamic bundle because of chronic pain. However, in this study, the thalamic microstructure was not altered on diffusion-weighted imaging.

The observed decrease in rT1/T2 signal in part of the thalamus could hypothetically also be an argument against a neuroinflammatory component, which has been described in previous experiments [24]. In neuroinflammation, macrophages and microglia play a crucial role. Resident microglia become active, and peripheral macrophages are recruited into the central nervous system. Although the characteristics of microglia and peripheral macrophages are highly idiosyncratic, a simplified dichotomous model of inflammatory/classical (M1)- and resolution/alternative (M2)-activated microglia/macrophages has been widely adopted [25]. Iron plays an important role in modifying the M1 phenotype of microglia and infiltrating macrophages [26], which explains why such neuroinflammatory processes can be detected by MRI techniques [20,27]. However, as we found a decrease in rT1/T2 signal in the PSP group compared to controls, the present study results could suggest indirect evidence against a neuroinflammatory component. Nonetheless, this interpretation remains indirect, and dedicated biomarkers, such as translocator protein (TSPO) PET, are required to conclusively assess the role of neuroinflammation in PSP.

It is widely accepted that the thalamus plays a key role in pain processing. More specifically, the lateral and medial nuclear groups are the thalamic subregions associated with the processing of noxious stimuli [28]. These nuclear groups correspond to the two distinct, but interrelated pathways of pain: the lateral and medial pathways. It is believed that the lateral pathway is more associated with the “sensory painfulness component”, whereas the medial pathway encodes the “suffering component” [29]. This current study suggests a decrease in microstructural tissue integrity in the medial thalamus. However, psychological comorbidities like depression and anxiety are highly prevalent in the chronic pain population [30], which might alter the affective evaluation of (chronic) pain. Affective psychiatric diseases are also known to induce structural changes and alter the rT1/T2w signal [23,31,32]. However, the present study did not account for variations in emotional states, such as by using questionnaires as a covariate. Additionally, the significant cluster in the thalamus also overlaps with the pulvinar, which is not commonly part of one of the pain pathways. Neuroimaging studies investigating trigeminal neuralgia, however, found decreased inferior and lateral pulvinar volumes [33] and functional alterations related to the pulvinar [34]. Furthermore, the involvement of the pulvinar in proprioception and multisensory processing has been described in primates [35], suggesting a role in pain processing. However, future research is needed to investigate this.

### 3.2. Evidence for Microstructural Alterations in the Dorsal Cervicomedullary Junction

In this study, we found evidence of a decrease in microstructural integrity within the dorsal cervicomedullary junction. Not only is the dorsal column a common target for spinal cord stimulation [36] but it has also been suggested to play a role in visceral pain [37]. Additionally, it is known to convey gnostic sensory information, such as fine touch, vibration, and proprioception, which are commonly impaired in stroke patients [38]. Further research is needed to determine the underlying mechanism of this finding and whether it relates to impaired gnostic sensibility, noxious stimuli, or both.

### 3.3. Evidence for Hypothetical Microstructural Changes in PSPS-II Patients

No significant changes in the rT1/T2 signal were observed when comparing the PSPS-II group with controls. Microstructural changes have been studied more widely in spinal pain syndromes. Multiple studies utilized DTI in patients with neuropathic pain after spinal cord injury and found significant microstructural changes in white matter tracts [39,40].

The findings of this study construct the hypothesis of differences in neurotransmitter receptor density loss. First, in the PSPS-II patients, the microstructural alterations that occurred were subtle compared to the microstructural organization of the brains of healthy controls. Second, the microstructural changes in PSP patients were more advanced compared to healthy controls and PSPS-II patients. One finding that supports this hypothesis is Spearman’s correlations per receptor atlas across both groups of chronic pain patients, depicted here as a radar plot in panel A. Panel C provides cross-sectional MRI data at different z-coordinates in MNI space and provides insights into the significant clusters where the PSP group exhibited lower rT1/T2 signal compared to controls. Notably, these clusters disappeared when comparing the PSP group with the PSPS-II group, which together form another finding supporting the theory of varying degrees of microstructural alterations in different chronic pain conditions. Panel B provides a visual representation of a slope of microstructural change in different disorders with various detection thresholds for different imaging techniques, which influences the potential of an imaging biomarker in different chronic pain disorders.

**Figure 3:**
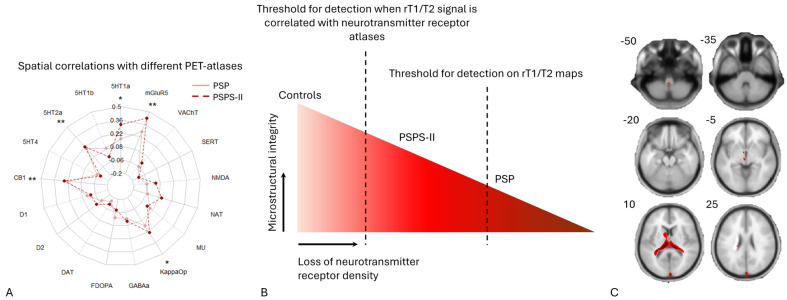
Visual hypothesis of varying degrees of microstructural degradation in different chronic pain syndromes. The findings of this study construct the hypothesis of differences in neurotransmitter receptor density loss. First, in PSPS-II patients, the microstructural alterations which occur are subtle as compared to the microstructural organization of the brain of the healthy controls. Second, the microstructural changes in PSP patients are more advanced as compared to healthy controls and PSPS-II patients. One finding that supports this hypothesis is the Spearman’s correlations per receptor atlas across both groups of chronic pain patients, depicted here as a radar plot in (**A**). (**C**) provides cross-sectional MRI data at different z-coordinates in MNI space and provides insights into the significant clusters where the PSP group exhibited lower rT1/T2 signal compared to controls. Notably, these clusters disappeared when comparing the PSP group with the PSPS-II group, which together form another finding supporting the theory of varying degrees of microstructural alterations in different chronic pain conditions. (**B**) provides a visual representation of a slope of microstructural change in different disorders with various detection thresholds for different imaging techniques, which influences the potential of an imaging biomarker in different chronic pain disorders. * = significant correlation in PSPS-II group, ** = significant correlation in both PSPS-II and PSP.

However, these studies examined spinal pain of sole neuropathic origin, whereas PSPS-II patients are known to have different somatosensory profiles [41]. Additionally, a combination of neuropathic, nociceptive, and nociplastic pain has been described in chronic low-back pain patients [42]. This could imply that sole neuropathic pain might induce greater central microstructural plasticity compared to pain of nociceptive or nociplastic origin. Furthermore, given that it is plausible to assume that the original lesion responsible for chronic back pain lies in the spinal cord, microstructural adaptation might occur more locally first. Further studies could investigate these hypotheses by studying rT1/T2 maps in pain syndromes of sole neuropathic origin and/or examining the rT1/T2 signal at the level of the spinal cord in PSPS-II patients. The lack of significant rT1/T2 findings in PSPS-II patients combined with the significant correlations between the rT1/T2 signal and the neurotransmitter receptor maps in the PSPS-II group might be explained by a different degree of microstructural alteration than within the PSP group (Figure 3). This hypothesis is derived from the finding that no significant clusters remained when comparing the PSPS-II group with the PSP group. If the microstructural integrity of the PSPS-II group were equal to the control group, similar clusters should have appeared when comparing the PSPS-II group with the PSP group. This observation might suggest that the microstructural alterations in the PSPS-II group may be in between on a gradual scale to those seen in the PSP group and the microstructural organization of the brain in healthy controls. However, the absence of significant rT1/T2 alterations in PSPS-II should be interpreted with caution. Importantly, the modest sample size for PSPS-II limits statistical power to detect subtle effects directly. Consequently, our results should be regarded as possible indirect evidence of possible microstructural alterations in PSPS-II. Further research should search for more sensitive imaging biomarkers to detect these hypothesized subtle microstructural alterations in PSPS-II patients.

### 3.4. Spatial Coupling of Microstructural Alterations with Neurotransmitter Systems

The observed spatial correlations between rT1/T2 maps and neurotransmitter receptor atlases in PSP and PSPS-II suggest that microstructural alterations in these chronic pain conditions may align with the spatial distribution of key neurochemical systems, including CB1, 5-HT1A/2A, mGluR5, and KappaOp receptors. These systems are known to be involved in pain modulation and perception and have been proposed as potential therapeutic targets [43,44,45,46]. However, the directionality of this relationship remains unclear. Alterations in rT1/T2 signal may reflect underlying changes in neurotransmitter systems, or conversely, the spatial distribution of these neurotransmitter systems may shape the pattern of microstructural alterations observed in disease. These findings point to a potential link between microstructure and neurotransmitter systems that warrants further investigation.

### 3.5. Strengths and Limitations

This study investigated the utilization of the rT1/T2 signal in patient cohorts suffering from chronic neuropathic pain. This study contributes a novel perspective to the field of neuroimaging biomarkers in chronic pain, as we are the first to utilize the rT1/T2 signal in this context. The analysis leveraged clinical data routinely acquired in the clinic. This could be considered both a strength and a limitation. While aligning with the envisioned application of the rT1/T2 signal as a supplementary biomarker without the need for additional MRI scans, the use of clinical data meant lower spatial resolution compared to data acquired solely for scientific purposes, limiting precision and detail. Additionally, the relatively small sample of our study may hinder the generalizability of our findings and warrants future investigation in a larger, prospective cohort. Furthermore, this limited sample size precluded the inclusion of covariates such as pain duration and emotional states in our analysis. Lastly, while our control group exhibited no structural abnormalities, these individuals were initially referred for imaging due to suspected unruptured aneurysms. This recruitment method may introduce a degree of selection bias, as hospital-referred individuals may not completely represent the general healthy population. However, all images were read by board-certified neuroradiologists in the clinical setting and reread for this study by a board-certified radiologist (D.H.) to ensure no structural abnormalities of the brain parenchyma were visible. Therefore, this limitation is believed to have had only a minor impact on the study outcomes.

## 4. Materials and Methods

### 4.1. Ethics Approval

Due to the retrospective nature of this study, ethics approval was waived by the local institutional review board. All patient data were anonymized to ensure confidentiality and compliance with data protection regulations.

### 4.2. Data and MRI Processing

Retrospective inclusion of patients suffering from PSPS-II or PSP was carried out by two of the researchers (R.W. and D.J.H.A.H.). Patients (≥18 years old) were included if MRI data containing both T1w and T2w images were available. All brain MRI scans (both T1-weighted and T2-weighted) were acquired on a 3.0-Tesla clinical scanner using standard clinical protocols within the same institution. Patients were excluded if the MRI data showed image artefacts (e.g., blooming artefacts due to foreign bodies such as bone conduction devices or severe motion artefacts). Particularly in the group of patients suffering from PSP, parenchymal defects were present. These lesions (e.g., lacunar infarctions, cortical infarctions, and adjacent regions of gliosis) were manually segmented using ITK-SNAP (version 4.0.1) by one of the researchers (D.J.H.A.H.; 9 years of experience in experimental neuroimaging). Additionally, a control cohort of healthy subjects without pain was included. This cohort comprised individuals who underwent an MRI examination due to a suspected unruptured cerebral aneurysm detected via CT imaging. Patients were included as control subjects if the MR imaging revealed no cerebral aneurysm, other brain pathology, or significant imaging artefacts.

**Figure 4:**
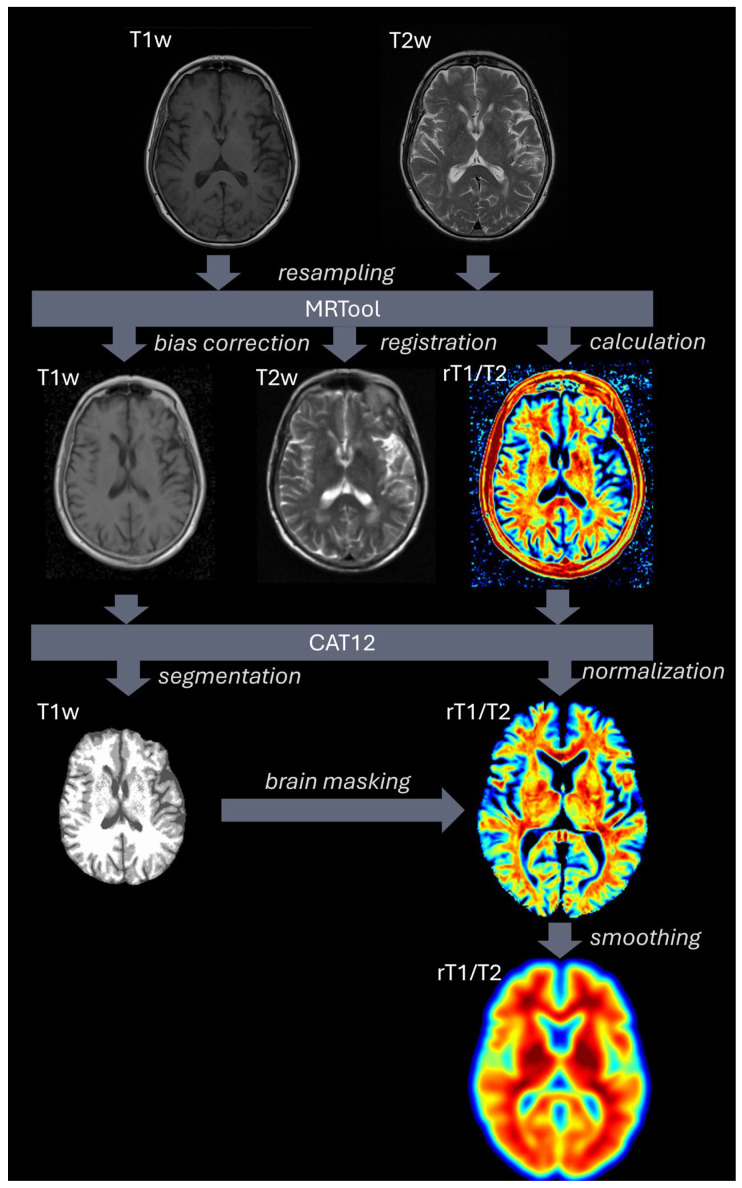
Overview of the image processing pipeline.

The process of generating and analyzing rT1/T2 images is based on SPM12 (v7771, Wellcome Centre for Human Neuroimaging, University College London, London, UK, http://www.fil.ion.ucl.ac.uk/spm, accessed on 10 April 2024) in MATLAB (vR2023a, MathWorks, Natick, MA, USA). All T1w and T2w data were converted from DICOM to Niftii using dicom2niftii (https://github.com/icometrix/dicom2nifti, accessed on 10 April 2024) and visually inspected for artefacts. To improve registration, the origins of all images were manually moved to the anterior commissure. Images were then resampled to an isotropic voxel size of 1 mm using 4th-degree B-spine interpolation. MRTool (version 1.4.3) [19,47], implemented in SPM12, was used for basic image processing and calculation of the rT1/T2 maps. In short, this toolbox rigidly registers the T2w image to the T1w image and corrects for image inhomogeneity. Thereafter, the signal intensities are standardized using a nonlinear histogram matching technique using the cerebrospinal fluid, skull bone, and surrounding soft tissues. All correction parameters were left to default [19,47]. Thereafter, the T1w images were segmented and spatially normalized to MNI space using the CAT12 toolbox (version 12.9) [48]. This toolbox utilizes an accurate DARTEL approach [49,50], which improves data normalization. After normalization, brains were extracted using masks generated by the Segment function of CAT12. Subsequently, the generated deformation matrices and brain masks were applied to the rT1/T2 maps. Furthermore, the segmentations of parenchymal defects were used as exclusion masks. These regions were not included in the data analysis. The final step concerned smoothing of all images with a 10 mm FWHM kernel. Please see Figure 4 for a schematic overview of these processing steps. Significant clusters were visualized using the xjView toolbox (https://www.alivelearn.net/xjview, accessed on 20 April 2024) and the BrainNet viewer [51].

T1w and T2w images were resampled to 1 mm isotropic resolution and processed using MRTool. This included bias correction of the T1w image, rigid registration of the T2w image to T1w space, and calculation of voxel-wise rT1/T2 ratio maps. Segmentation and spatial normalization of T1w images to MNI space were performed using CAT12. Brain masks derived from segmented T1w images were applied to the normalized rT1/T2 maps. Finally, all rT1/T2 maps were smoothed using a 10 mm FWHM Gaussian kernel.

### 4.3. Spatial Correlation of rT1/T2 Signal with Neurotransmitter Maps

For this analysis. the JuSpace toolbox (version 1.5) implemented in MATLAB (https://github.com/juryxy/JuSpace/tree/JuSpace_v1.5, accessed on 19 May 2024) was utilized. This toolbox enables the spatial correlation of different imaging modalities with neurotransmitter atlases derived from molecular imaging techniques (i.e., positron-emission tomography (PET) and single-photon-emission computed tomography (SPECT)). Specifically, the toolbox parcellates the MRI data and selected neurotransmitter atlases into the 119 regions delineated by the Neuromorphometrics atlas. Subsequently, the toolbox extracts the mean intensity values from these regions and performs correlation analyses between rT1/T2 maps and the neurotransmitter atlases. Individual z-score maps for whole-brain rT1/T2 images of PSP and PSPS-II patients were calculated by use of the whole-brain rT1/T2 images of the healthy control group. These z-score maps were then subjected to Spearman correlation analysis with the all neurotransmitter atlases within the JuSpace toolbox: (a) 5-hydroxytryptamine receptor subtypes 1a, 1b, 2a, and 4 (5HT1a, 5HT1b, 5HT2a, 5HT4); (b) cannabinoid receptor 1 (CB1); (c) dopamine receptor subtypes 1 and 2 (D1, D2); (d) dopamine transporter (DAT); (e) fluorodopa (FDOPA); (f) gamma-aminobutyric acid type a receptor (GABAa); (g) kappa opioid receptor (KappaOp); (h) mu-opioid receptor (MU); (i) norepinephrine transporter (NAT); (j) N-methyl-D-aspartate receptor (NMDA); serotonin transporter (SERT); (k) vesicular acetylcholine transporter (VAChT); and l) metabotropic glutamate receptor 5 (mGluR5). If multiple atlases were available for a specific receptor (system), the atlas derived from the largest sample of healthy controls was used for correlation statistics. Moreover, this analysis was adjusted for spatial autocorrelation using the gray matter tissue probability map from SPM12. Exact permutation-based *p*-values were calculated using 10,000 permutations and were corrected for multiple comparisons using the false-discovery rate (FDR) method.

### 4.4. Statistical Analyses

Descriptive statistics were analyzed using RStudio (version 2023.09.1+494). The normality of the data was tested using the Shapiro–Wilk normality test to determine the appropriate statistical tests for subsequent analyses. For age, when normally distributed, an independent *t*-test was used. For non-normally distributed data, the Mann–Whitney U test was used. To assess differences in sex distribution, Fischer’s exact test was utilized.

Whole-brain analyses were conducted in SPM12 (v7771, Wellcome Centre for Human Neuroimaging, University College London, London, UK, http://www.fil.ion.ucl.ac.uk/spm http://www.fil.ion.ucl.ac.uk/spm, accessed on 10 April 2024) using an independent two-sample *t*-test design [52,53], with a cluster threshold of 750. Significance was determined based on *p*-values, with thresholds set to *p* < 0.05 for descriptive statistics and *p* < 0.001 for whole-brain analysis.

## 5. Conclusions

The presented study utilized the rT1/T2 signal as a biomarker for tissue integrity and neuroinflammation in patients with PSP and PSPS-II. Significant reductions in rT1/T2 signal were observed in the dorsal and medial thalamus, cuneus, and dorsal cervicomedullary junction in PSP patients, indicating decreased microstructural integrity. No similar changes were observed in PSPS-II patients. Spatial correlation analysis with neurotransmitter maps revealed significant correlations in both patient groups. However, given the small sample of this pilot study, further research with larger cohorts is necessary to confirm these results and clarify the role of microstructural changes in chronic pain. Future studies should also explore the rT1/T2 signal in other pain syndromes and at the spinal cord level to clarify the role of microstructural changes in chronic pain and enhance the understanding of pain pathophysiology.

## Figures and Tables

**Table 1:** Demographic characteristics.

Group	Sample Size (n)	Age (at Onset) in Years	Number of Males/Females
PSP	11	64.3 (± 12.5)	4/7
PSPS-II	15	57.9 (± 9.7)	8/7
Controls	18	60.5 (± 13.0)	9/9

**Table 2:** The peak and gravity MNI coordinates of the significant regions after cluster thresholding.

Cluster	Peak MNI			Centre of Gravity MNI			Number of Voxels	Best Corresponding Location
	**x**	**y**	**z**	**x**	**y**	**z**		
1	−3	−47	−57	−1	−47	−61	1138	Cervicomedullary junction/caudal medulla (bilateral)
2	−10	−20	14	−8	−23	12	17533	Thalamus (bilateral) and caudate nucleus (left)
3	3	−100	5	2	−97	13	1522	Occipital lobe/cuneus (bilateral)
4	−28	−4	71	−27	0	73	1333	Superior frontal gyrus (left)

**Table 3:** Statistical metrics of spatial correlation between rT1/T2 signal intensity and receptor atlases.

Receptor Atlas	PSPS-II		PSP	
	Mean Fisher’s z (Spearman’s rho)	FDR-Corrected *p*-Value	Mean Fisher’s z (Spearman’s rho)	FDR-Corrected *p*-Value
5HT1a	**0.3116**	**1.70 × 10^−3^**	0.1581	0.117572
5HT1b	−0.0045	0.94431	0.0886	0.33736
**5HT2a**	**0.221**	**1.70 × 10^−3^**	**0.1933**	**0.027197**
5HT4	−0.1018	0.1491	−0.0646	0.539189
**CB1**	**0.2537**	**0.00255**	**0.2234**	**0.002833**
D1	−0.0138	0.94431	0.0264	0.853679
D2	−0.0221	0.853679	−0.0901	0.456083
DAT	−0.12	0.113424	−0.1545	0.125525
FDOPA	−0.0853	0.349784	−0.0048	0.94431
GABAa	0.0373	0.652741	0.0049	0.94431
KappaOp	**0.2339**	**0.00272**	0.1512	0.080573
MU	0.0078	0.94431	0.0483	0.739996
NAT	0.1068	0.229054	−0.0436	0.853679
NMDA	0.0328	0.798782	−0.0656	0.635897
SERT	−0.13	0.067237	−0.1257	0.162957
VAChT	−0.0073	0.94431	−0.06	0.643311
**mGluR5**	**0.4265**	**0.002266**	**0.2749**	**0.033572**

Exact mean Fisher’s z in Spearman’s rho and permutation-based *p*-values after FDR correction calculated by the JuSpace toolbox when correlating rT1/T2 signal with various receptor atlases derived from molecular imaging techniques. Values are provided for both included groups of chronic pain patients. Significant correlations are shown in bold.

## Data Availability

The datasets generated and analyzed during the current study are available from the corresponding author on reasonable request. The data are not publicly available due to their containing information that could compromise the privacy of the participants.
